# Model construction of medical endoscope service evaluation system-based on the analysis of Delphi method

**DOI:** 10.1186/s12913-020-05486-x

**Published:** 2020-07-09

**Authors:** Jun Zheng, Ligang Lou, Ying Xie, Siyao Chen, Jun Li, Jingming Wei, Jingyi Feng

**Affiliations:** 1 The First Affiliate Hospital of Medical College, Zhejiang University, Hangzhou, 310003 China; 2 Faculty of Business and Law, Anglia Ruskin University, Bishop Hall Lane, Chelmsford, CM1 1SQ United Kingdom; 3 Center for Health Policy Studies Zhejiang University School of Medicine, Hangzhou, 310058 China

**Keywords:** Medical endoscope, Evaluation index system, Delphi method

## Abstract

**Background:**

Medical endoscope is widely used in clinical practice for the purpose of diagnosis and treatment, occupying around 5% of the medical device market. Evaluating the true service level of medical endoscope is essential and necessary to improve overall performance of medical diagnosis and treatment, and to maintain competitiveness of endoscope manufacturers, however, such a tool is not available in the market. This study develops an Evaluation Index System (EIS) to assess service level of medical endoscope, and to provide suggestions for improving the service level through the Delphi method.

**Methods:**

Firstly, the possible factors influencing the service level were identified from literature review. In parallel, the Delphi expert method questionnaire was designed and 25 experts were invited to conduct three rounds of questionnaire, to evaluate and rate the possible factors. Finally, we determined the weights associated with the factors, using the analytic hierarchy process (AHP) and percentage method, and developed the service level EIS.

**Results:**

The EIS consists of 3 first-level indicators, 24 s-level indicators and 68 third-level indicators. According to the weights computed using AHP, first-level indicators are ranked as post-sale (0.62), in-sale (0.25) and pre-sale (0.13). Through case verification, the medical endoscope brand Olympus had a total score of 4.17, Shanghai Aohua had a total score of 3.71, and Shanghai Chengyun had a total score of 3.28, which matches its market popularity and ranking in terms of market share. The results obtained from the EIS are consistent with the reality.

**Conclusions:**

The EIS established in this study is comprehensive, reliable and reasonable with strong practicality. The EIS can act as a tool for the endoscope users to evaluate potential products and make informed choices. It also provides a measurable basis for endoscope manufacturers and service providers to improve service quality.

## Background

Medical endoscope is an important surgical equipment for minimally invasive treatment technology, which has the advantages of small trauma, short operation time and quick postoperative recovery and plays an important role in surgery and operation [1–4]. For a long time, imported medical endoscopes have been more favorable due to perceived higher variety, better quality, more advanced technology and better service. With the extra investment in medical technology research and development, medical endoscopes produced by domestic manufacturers in China are quickly catching up in terms of product variety, quality, performances and innovation. Medical devices such as endoscopes are not a one-off purchase, instead, the associated post-sale service provided by the manufacturers or qualified dealers plays an important role in influencing decision making by the clients. Medical endoscopes are widely used for medical diagnosis and treatment, hence, the registration, the administration and the service level of endoscopes have received attention from the top level Ministry of Health in China, mid level Chinese State Administration of Food and Drug, and various medical institutions and medical device manufacturers. In this study, we measure the service level of medical endoscope from the perspectives of producers and end users. Medical institutions purchase endoscopes and professional doctors with professional licenses become the end users. Since medical endoscopes are applied to patients, medical endoscopes not only require strict disinfection, inspection and storage before and after use, but also regular quality and safety inspections by engineers [5, 6]. Currently there is no evaluation standard available to assess the service level provided by medical endoscope suppliers. The huge variety of medical endoscopes, in terms of brands, types, functions and application scenarios, makes the evaluation more challenging [7–10]. Most of the studies to date only evaluated the post sales service of medical endoscope suppliers, for example, 6 indicators were agreed to assess post sale service of medical devices used in China [11], and similarly 16 indicators were identified by Shanghai Sixth People’s Hospital using Delphi method to assess post sale service [12]. These evaluation systems did not consider pre-sale or during sale service, nor did the systems make comparison between different brands, which are all important factors influencing decision making when end users choose endoscopes.

As a subjective and qualitative method, Delphi method produces reliable results and draws unified conclusions from sufficient data, which provides a strong foundation to identify key indicators that are used to construct an EIS for medical endoscope. In this research, we use Delphi method to establish a comprehensive Evaluation Index System (EIS) to assess service level across the life cycle of a medical endoscope, staring from pre-sale, to in-sale and ending with post-sale. The weights of various indicators in the evaluation index are determined using AHP and percentage method [13–15]. The new EIS provides a tool to evaluate service level of medical endoscope, strengthening the quality control in manufacturers or service providers and enabling users to make informed decision.

## Methods

### An overview of Delphi method

The Delphi method was originally conceived to study an Air Force-sponsored Rand Corporation, to obtain the most reliable consensus of opinion of a group of experts [16]. In its infancy Delphi is characterized as a method for structuring effective communication process to allow a group of individuals to reach a group consensus. Nowadays the Delphi method has evolved to become a fundamental tool in the areas of forecasting, evaluation and concept/framework development, when there is a need to incorporate subjective information directly into evaluation models. Traditional Delphi method consists of six phases [17, 18]: (1) appoint a group facilitator who selects a group of experts based on the topic being examined; (2) identify experts and assemble expert panel; (3) define problem and develop questionnaire; (4) brainstorm alternatives through Round 1 questionnaires; (5) analyze, summarize and narrow alternatives through controlled feedback; and (6) rank alternatives in subsequent rounds of questionnaires and reach a closer consensus. At the end of each round of questionnaire, all questionnaires are returned to the facilitator who decides if another round is necessary or if the results are ready to support decision making. The questionnaire rounds can be repeated as many times as necessary to achieve a general sense of consensus [19, 20].

### Research using Delphi method

#### Phase 1 appointing a group facilitator

The endoscopic service level research group appointed a team leader to setup the expert group. This team leader specializes in medical equipment management and maintenance management.

#### Phase 2 identifying experts and assembling expert panel

##### Selection criteria

This study adopted the method of non-probability subjective sampling and appointed experts from medical institutions or medical device enterprises who meet the following criteria:

(A) working in medical institutions, and engaging with medical endoscope application, including medical doctors, medical device engineers;
(B) working in medical device enterprises, associated with the production, sale and post-sales service of medical endoscopes
(C) having a professional title at advanced level or above
(D) having a positional title at middle level or above
(E) having more than 5 years’ working experience in the positions outlined in (A-D).

##### Degree of expert authority

In addition to selection criteria outline in (A-E) above, degree of expert authority Cr is introduced to add or remove experts from each round of questionnaires. The degree of expert authority Cr is defined using two self-evaluation scores that were given by the experts in each round of questionnaire, to reflect the reliability of experts’ opinions:

1 $$ \mathrm{Cr}=\left(\mathrm{Ca}+\mathrm{Cs}\right)/2 $$

where Cs is expert’s knowledge base to judge the program, and Ca is the expert’s familiarity with the problem. Cs and Ca range between 0 and 5, with a higher value indicating more reliable judgment and more familiarity with the problem [16] (see Table 1). If the self rated Cr is higher than the threshold 2, the expert is kept, otherwise the expert is removed from group.

**Table 1:** Quantitative self-evaluation scores Cs and Ca

Judgement basis (Cs)	Quantitative value (score)	Familiarity (Ca)	Quantitative value (score)
Practical experience	5.0	Very familiar	5.0
Theoretical analysis	4.0	Familiar	4.0
Understanding of relevant progress at home and abroad	3.0	General familiarity	3.0
Reference	2.0	Not very familiar	2.0
Subjectivity	1.0	Unfamiliar	1.0

In the first round, following selection criteria (A-E) we chose 30 medical endoscope managers from medical institutions and endoscope enterprises to participate. In the second round, degree of expert authority was computed to filter the experts, with only 15 eligible experts remained. Ten new experts from medical institutions were invited to participate the second round of questionnaire, making the total number of participants as 25. The same procedure was applied to the third round, which consisted of 20 medical endoscope managers from medical institutions. The reduced number of samples in each round was in accordance with the correlation function, which satisfied gender and age diversity, as shown in Table 2.

**Table 2:** Basic situation of the sample

	Unit		Working years			Professional title	Positional title
	Hospital	Enterprise	5–10 years	11–20 years	More than 20 years	Advanced or above	Middle or above
**First round**	15	15	15	2	13	13	17
**Second round**	25	0	0	5	20	25	23
**Third round**	20	0	0	2	18	20	20

#### Phases 3 define problem and develop questionnaire

A comprehensive review on medical endoscope development is conducted by extracting, analyzing and comparing findings from previous studies. We also searched Pubmed, Web of Science, cnki.net, Wanfang database and other databases to understand the status quo of medical endoscopy service evaluation and analyzed the factors that affect the evaluation of medical endoscope services, which formed the theoretical basis for this research. This study used Nvivo 8.0 software coding function, and micro-analysis of the literature, to establish preliminary categories and sub-projects.

**Fig. 1:**
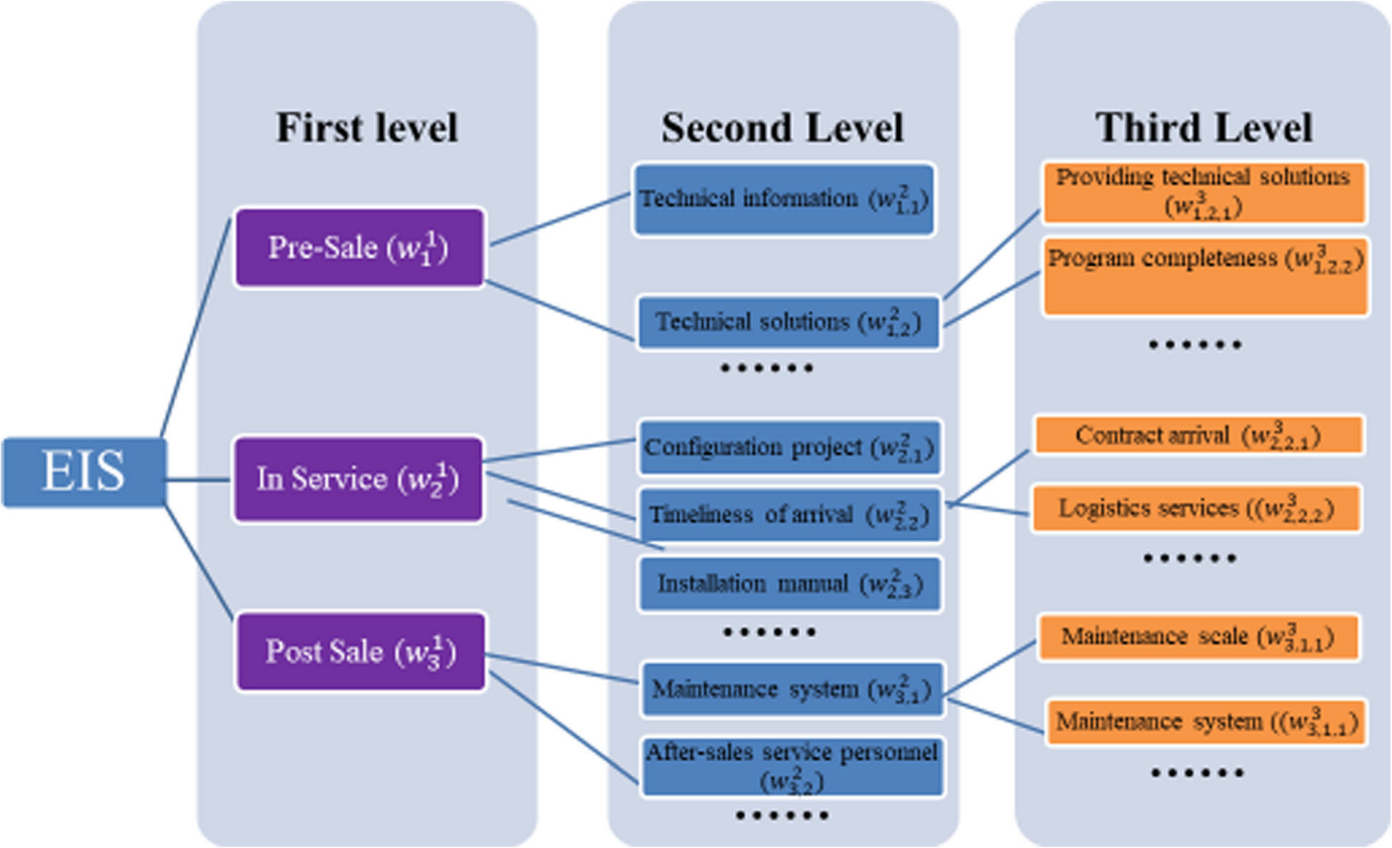
Hierarchical structure of indicators at three levels

Based on the aforementioned work, a three-level EIS is constructed to assess the service level of medical endoscope, consisting of the First Level, the Second Level and the Third Level (see Fig. 1).

As shown in Fig. 1, there are three categories at the First Level, including pre-sale, in-sale and post-sale. At the second level a group of sub-indicators are identified, and more sub-indicators are included at the Third Level. These indicators formed a basis to develop the first round of questionnaire that seeks to measure perceived importance score rated by experts for each indicator (see Appendix 1 for a sample of the questionnaire which shows all the questions of Level 1 and 2 indicators, and part of the questions of Level 3 indicators). The indicators were rated on a Likert scale of 1–5, where 5 = Very important and 1 = Not important.

#### Phases 4–6 three rounds of questionnaires and rank alternatives

The first round of expert consultation focused on constructing a hierarchical structure of medical endoscope service level EIS. The first round of questionnaire was issued to the selected experts by post, together with the instructions to fill the questionnaire based on their personal opinion, experience or pervious research. Based on the importance scores and feedback received in the first round, we revised the list of medical endoscope service level indicators by removing the indicators that did not meet a set of criteria, clustering similar indictors, and adding new indicators that were missed.

The second round and third round of expert consultation mainly focused on the repeated rating of the importance scores of the indicators. In the third round, experts were also invited to determine the weights of all the remaining indicators, with the AHP procedure adopted to rank the three indicators at the First Level, and the percentage method applied to rank the indicators at the Second and Third Levels.

##### Computing indicators’ weights using AHP and percentage method

The weight assigned to each of the three indicators at the First Level is equal to the sum of percentages of sub-indicators descended from that indicator, as shown in eqs. (2) and (3) (see Fig. 1):

2 $$ {W}_k^n={\sum}_{i=1}^m{W}_{k,i}^{n+1} $$

3 $$ {W}_{k,i}^{n+1}={\sum}_{j=1}^p{W}_{k,i,j}^{n+2} $$

Where *n* represents the level, i.e., the First, Second or Third level; *k* is the *k*th indicator at the *n*th level; *i* is the *i*th sub-indicator descended from the *k*th indicator at the *n*th level (*i = 1,2,…m*); *j* is the *j*th sub-indicator descended from the *i*th indicator at the *(n + 1)* th level. Through two rounds of expert consultation, the feedback information of experts was analyzed and the evaluation indicators were revised twice.

The medical endoscope service level EIS and the weight of each indicator was developed using three rounds Delphi method, AHP and percentage method [16, 21]. The index score of a medical endoscope was calculated as the aggregate importance score of all the indicators, i.e., the weighted sum of importance scores of individual indicators.

##### Quantitative criteria for inclusion and deletion of indicators

The concentration of expert opinions is mainly determined by the Average Score (M) and the Full Score Frequency (K), which reflect the importance of the indicator in evaluating medical endoscope service level. M is the mean value of the importance scores rated by all the experts, and K is the frequency of receiving full scores (rating 5 in this study) from experts. “Q_+_” indicates the maximum value of the expert’s importance score, “Q_−_” indicates the minimum value of the expert’s score, and Q_+_-Q_−_ indicates the extreme value of the expert index score. The smaller the extreme value, the higher the concentration, when Q_+_-Q_−_ < 2, it indicates that the concentration of expert opinions is good.

Following the Delphi method, a quantitative assessment was conducted to screen the indicators to be included in the EIS. To include an indicator in the EIS, three criteria must all be met: (1) Full Score Frequency K is above the critical value of K = 0.3; (2) the mean value of importance score M is higher than the critical value of M = 4; and (3) the extreme value Q_+_-Q lower than or equal to the critical value of Q_+_-Q_−_ = 2.

If one or two criteria are not met, a discussion is required with experts to decide whether to cluster the indicator with others or delete it.

Hence, when finalizing the indicators to be included in the EIS, an aggregate decision is drawn upon expert suggestions, importance scores of indicators obtained by Delphi method, and the analysis and discussion of the research group [22].

## Results

### Expert positive coefficient

The expert positive coefficient is the degree of attention and interest of experts in the research. In this study, a total of 3 rounds of expert consultations were conducted, as shown in Table 3. In the first round, 30 questionnaires were issued, and 25 were collected, of which 18 were valid questionnaires, and the expert positive coefficient was 83.3%. In the second round, 25 expert consultation forms were issued, and 20 were collected. Among them, 19 were valid questionnaires, so the expert positive coefficient was 80.0%; in the third round 20 questionnaires were issued, 20 were recovered, of which 19 were valid questionnaires, and the expert positive coefficient was 100.0%. From the statistical results of the three rounds of experts’ positive coefficient, most experts had a higher level of participation in this study.

**Table 3:** Expert positive coefficient

	First round	Second round	Third round
Issued	30	25	20
Received	25	20	20
Expert positive coefficient	83.3%	80.0%	100.0%

### Reliability analysis of questionnaire design

Cronbach’s α is a statistic value, referring to the average value of half reliability coefficient obtained by all possible item partitioning methods of the scale. The value of Cronbach’s α coefficient is between 0 and 1, and a value above 0.8 indicates good reliability of the scale. The computed reliability coefficient of the questionnaire is α = 0.976, which showed that the questionnaire was well designed and had high reliability [23–25].

### Degree of concentration of expert opinions

As an example, the degree of expert opinion concentration is calculated for some of the indicators at the Second Level, as shown in Table 4. The Mean Scores M of technical solutions, device installation and maintenance system were more than 4; the Full Score Frequency K was more than 0.3 for each indicator; and the difference of extreme value Q_+_-Q_−_ was majorly less than or equal to 2, which indicated that experts’ opinions were well concentrated. However, the difference of extreme value of complaint handling was more than 2, which indicated that the concentration of expert opinions was slightly poor.

**Table 4:** Degree of concentration of expert opinions

Indicators	M	K	Q + -Q-	extreme value difference
1.3 Technical solutions	4.429	0.524	2	≤2
2.4 Device installation	4.857	0.857	1	≤2
3.1 Maintenance system	4.571	0.619	2	≤2
3.7 Maintenance response	4.810	0.810	1	≤2
3.16 Maintenance quality	4.619	0.476	1	≤2
3.3 Complaint handling	4.048	0.333	4	> 2
…	…	…	…	…

### Screening results of medical endoscope service level indicators

In the first two rounds of questionnaire, numerical results M, K and Q_+_-Q_−_ were calculated, to redefine and cluster the list of indicators before the third round questionnaire. Post the third round of questionnaire, quantitative criteria M, K and Q_+_-Q_−_ were applied to finalize the list of indicators, with the indicators with modified twice and lower importance scores being deleted [26].

Based on a comprehensive review of the literature, and consideration of experts’ opinions, we selected 123 indicators to design the first round of questionnaire, including 35 indicators at the Second Level, and 88 indicators at the Third Level. After the first round of questionnaire, the numerical results of measuring the indicators against the three quantitative criteria were calculated, i.e., Average Score M, Full Score Frequency K, and extreme value difference Q_+_-Q_−_, to remove and add indicators to the Second and Third levels, as presented in Table 5.

**Table 5:** Quantitative measures in the first round

	Initial number of indicators	Average Score M > 4	K > 0.3	Q_+_-Q- < =2	Number of indicators at the end of first round
**Second level indicators**	35	32	33	23	34
**Third level indicators**	88	78	77	63	82

For example, the Average Score M of the indicator 3.18 “Function Development” was 3.553, the Full Score Frequency K was 0.222, and the extreme value difference Q_+_-Q was 5, which indicated that experts had a low degree of recognition of this indicator and the indicator should be removed. According to the results obtained from the first round of the Delphi method, 1 indicator at the Second Level was deleted, leaving 34 indicators at the Second Level. 6 indicators at the Third Level were deleted, 2 indicators at the Third Level were merged into one new indicator,1 new indicator for product trials was added to the Third Level, and 2 third-levels of indicators were redefined, leaving 82 indicators at the Third Level (see Table 5).

The quantitative measures of the indicators against the three criteria in the second round are presented in Table 6. According to the results of the second round of questionnaire, one indicator at the Second Level was deleted, and 4 indicators were merged into one new indicator, leaving 30 indicators at the Second Level. At the Third Level, 4 indicators were merged into 2 new indicators (among the four indicators, two indicators were merged into one), 7 were deleted, and 3 new indicators were added, leaving 76 indicators at the Third Level.

**Table 6:** Quantitative measures in the second round

	Initial number of indicators	Average Score M > 4	K > 0.3	Q_+_-Q- < =2	Number of indicators at the end of second round
**Second level indicators**	34	25	25	23	30
**Third level indicators**	82	76	61	63	76

Table 7 and Table 8 present the statistical results of each indicator at the Second and Third levels, including Average Score M, Full Score Frequency K, Extreme Value Q_+_-Q_−_, and Degree of Expert Authority Cr. These results were compared with the criteria (1)–(3) defined in the Section “**Quantitative criteria for inclusion and deletion of indicators”,** and the indicators were removed or retained accordingly based on the comparison outcome or experts’ judgement.

**Table 7:** Statistical results of second-level indicators identified under Delphi method

Second-level indicators	M	Standard deviation	Coefficient of variation	K	Q_+_-Q_−_	Cr
1.1 Product display	4.000	0.837	0.209	0.286	3	4.000
1.2 Technical information	4.429	0.676	0.153	0.524	2	4.476
1.3 Technical solutions	4.476	0.750	0.167	0.571	3	4.405
1.4 Requirement Demonstration	4.190	1.030	0.246	0.476	4	4.000
1.5 Sales system	3.143	0.573	0.182	0.000	2	3.119
1.6 New technology promotion	3.571	0.926	0.259	0.143	3	3.571
2.1 Configuration project	4.619	0.740	0.160	0.714	3	4.405
2.2 Timeliness of arrival	4.381	0.590	0.135	0.429	2	4.476
2.3 Installation manual	4.476	0.680	0.152	0.571	2	4.429
2.4 Device installation	4.857	0.359	0.074	0.857	1	4.524
2.5 Equipment commissioning and quality control	4.667	0.730	0.156	0.810	2	4.500
2.6 Equipment acceptance	4.810	0.402	0.084	0.810	1	4.643
2.7 Data protocol	4.190	0.873	0.208	0.476	2	4.048
2.8 Primary operational training	4.667	0.577	0.124	0.714	2	4.429
3.1 Maintenance system	4.571	0.598	0.131	0.619	2	4.714
3.2 Post-sales service personnel	4.571	0.746	0.163	0.714	2	4.500
3.3 Complaint handling	4.048	0.973	0.241	0.333	4	4.167
3.4 Adverse event monitoring	4.190	0.750	0.179	0.381	2	4.214
3.5 Product recall	4.000	0.949	0.237	0.381	3	3.952
3.6 Maintenance and use manual	4.714	0.644	0.137	0.810	2	4.429
3.7 Maintenance response	4.810	0.402	0.084	0.810	1	4.619
3.8 Maintenance accessories	4.714	0.463	0.098	0.714	1	4.619
3.9 Standby machine	4.381	0.669	0.153	0.476	2	4.405
3.10 Warranty contract	4.619	0.498	0.108	0.619	1	4.619
3.11 Maintenance and repair report	4.476	0.750	0.167	0.571	3	4.524
3.12 Retraining of clinical operations	4.238	0.831	0.196	0.476	2	4.214
3.13 Retraining in clinical application	4.286	0.784	0.183	0.476	2	4.119
3.14 Technical support	4.333	0.658	0.152	0.429	2	4.238
3.15 Scientific research cooperation	3.714	1.102	0.297	0.286	4	3.976
3.16 Maintenance quality	4.619	0.498	0.108	0.619	1	4.619

**Table 8:** Statistical results of third-level indicators identified under Delphi method

Third-level indicators	M	Standard deviation	Coefficient of variation	K	Q_+_-Q-
1.1.1 In-hospital training demonstration	3.952	0.805	0.204	0.238	3
1.1.2 Out-of-hospital training demonstration	3.476	1.078	0.310	0.143	4
1.2.1 Technical information	4.524	0.602	0.133	0.571	2
1.3.1 Providing technical solutions	4.619	0.740	0.160	0.714	3
1.3.2 Program completeness	4.381	0.865	0.197	0.571	3
1.4.1 Requirement Demonstration	3.952	1.161	0.294	0.429	4
1.5.1 Product category	3.333	0.730	0.219	0.095	3
1.6.1 Popularization and trial	3.714	0.902	0.243	0.190	3
2.1.1 Programme effectiveness	4.619	0.740	0.160	0.714	3
2.2.1 Contract arrival	4.571	0.598	0.131	0.619	2
2.2.2 Logistics services	4.000	0.949	0.237	0.381	3
2.3.1 Accompanying documentation	4.571	0.598	0.131	0.619	2
2.4.1 Device installation	4.857	0.359	0.074	0.857	1
2.4.2 Installation efficiency	4.190	0.814	0.194	0.381	3
2.4.3 Installation report	4.190	0.873	0.208	0.476	2
2.4.4 Installation service	4.286	0.717	0.167	0.429	2
2.5.1 Installation and commissioning	4.714	0.561	0.119	0.762	2
2.5.2 Quality inspection	4.333	0.966	0.223	0.619	3
2.5.3 Quality control record	4.571	0.870	0.190	0.762	3
2.5.4 Quality control service satisfaction	4.381	0.740	0.169	0.524	2
2.6.1 Acceptance process	4.714	0.561	0.119	0.762	2
2.6.2 Acceptance Time	4.238	0.944	0.223	0.476	3
2.6.3 Unacceptable processing	4.429	0.676	0.153	0.524	2
2.6.4 Acceptance service	4.429	0.676	0.153	0.524	2
2.7.1 Data opening	3.952	0.921	0.233	0.381	2
2.8.1 Normative training	4.381	0.740	0.169	0.524	2
2.8.2 Clinical training	4.524	0.512	0.113	0.524	1
2.8.3 Medical training	4.429	0.676	0.153	0.524	2
3.1.1 Engineer qualification	4.429	0.746	0.169	0.571	2
3.1.2 Maintenance scale	4.000	0.775	0.194	0.286	2
3.1.3 Maintenance and certification	4.095	0.768	0.188	0.333	2
3.1.4 Maintenance system	4.095	0.768	0.188	0.333	2
3.1.5 Maintenance implementation normative	4.476	0.750	0.167	0.619	2
3.1.6 Maintenance response time	4.905	0.301	0.061	0.905	1
3.1.7 Troubleshooting time	4.714	0.561	0.119	0.762	2
3.2.1 Post-sales team	3.905	0.700	0.179	0.190	2
3.2.2 Team training	4.429	0.746	0.169	0.571	2
3.2.3 Satisfaction with team service	4.524	0.602	0.133	0.571	2
3.2.4 Satisfaction with maintenance service	4.619	0.498	0.108	0.619	1
3.3.1 Complaint procedure	3.952	0.865	0.219	0.190	4
3.3.2 Complaint record	3.714	0.902	0.243	0.143	4
3.3.3 Complaint handling	4.095	0.995	0.243	0.381	4
3.3.4 Convenience of complaints	4.238	0.889	0.210	0.476	3
3.3.5 Complaint feedback	4.238	0.831	0.196	0.429	3
3.4.1 Adverse event monitoring	4.190	0.928	0.222	0.476	3
3.4.2 Report of adverse events	4.048	0.865	0.214	0.333	3
3.4.3 Adverse event handling	4.095	0.768	0.188	0.333	2
3.4.4 Adverse event record	4.429	0.676	0.153	0.524	2
3.5.1 Product recall	3.619	0.740	0.204	0.048	3
3.6.1 Operation manual	4.667	0.730	0.156	0.810	2
3.6.2 Service manual	4.524	0.814	0.180	0.667	3
3.6.3 Openness of technical data	4.429	0.746	0.169	0.571	2
3.7.1 PM program	4.571	0.598	0.131	0.619	2
3.7.2 Satisfaction with PM service	4.476	0.602	0.134	0.524	2
3.7.3 Satisfaction with maintenance hotline	4.143	0.727	0.175	0.333	2
3.7.4 Satisfaction of Maintenance Response	4.476	0.680	0.152	0.571	2
3.7.5 Satisfaction with troubleshooting	4.667	0.577	0.124	0.714	2
3.8.1 Quality of maintenance accessories	4.619	0.498	0.108	0.619	1
3.8.2 Speed of arrival of repair accessories	4.524	0.512	0.113	0.524	1
3.8.3 Satisfaction with maintenance price	4.571	0.598	0.131	0.619	2
3.8.4 Satisfaction with payment method	4.143	0.854	0.206	0.429	2
3.9.1 Whether to provide a standby machine	4.524	0.750	0.166	0.667	2
3.9.2 Satisfaction with standby service	4.333	0.730	0.169	0.476	2
3.10.1 Contract Integrity	4.524	0.602	0.133	0.571	2
3.10.2 Satisfaction with contract economy	4.476	0.512	0.114	0.476	1
3.10.3 PM Satisfaction in Contract	4.333	0.658	0.152	0.429	2
3.10.4 Satisfaction with contract indicators	4.429	0.676	0.153	0.524	2
3.10.5 Satisfaction with Contract evaluation	4.286	0.717	0.167	0.429	2
3.11.1 Satisfaction with reporting quality	4.524	0.512	0.113	0.524	1
3.11.2 Satisfaction with report completion rate	4.333	0.577	0.133	0.381	2
3.12.1 Satisfaction with operational retraining	4.190	0.814	0.194	0.429	2
3.13.1 Satisfaction with application retraining	4.286	0.717	0.167	0.429	2
3.14.1 Technical support	4.143	0.573	0.138	0.238	2
3.15.1 Scientific research cooperation	3.714	1.056	0.284	0.238	4
3.16.1 Probability of the same fault occurrence	4.524	0.602	0.133	0.571	2
3.16.2 Satisfaction with equipment Performance	4.476	0.512	0.114	0.476	1

In the third round of questionnaire, the number of deleted indicators at the Second and Third Levels were 6 and 8 respectively, keeping 24 indicators at the Second Level and 68 indicators at the Third Level (see Table 9). Following the three rounds of questionnaire, we constructed an EIS, including 3 indicators at the First Level, 24 indicators at the Second Level, and 68 indicators at the Third Level [27].

**Table 9:** Weighting of indicators using AHP and percentage method

First-level categories	Weight	second-level indicators	Weight	Third-level indicators	Weight
Pre-sale service	0.1345	1.1 Technical information	0.0669	1.1.1 Technical information	0.0669
		1.2 Technical solutions	0.0676	1.2.1 Providing technical solutions	0.0347
				1.2.2 Program completeness	0.0329
Sale service	0.2568	2.1 Configuration project	0.0324	2.1.1 Programme effectiveness	0.0324
		2.2 Timeliness of arrival	0.0307	2.2.1 Contract arrival	0.0164
				2.2.2 Logistics services	0.0143
		2.3 Installation manual	0.0314	2.3.1 Accompanying documentation	0.0314
		2.4 Device installation	0.0340	2.4.1 Device installation	0.0094
				2.4.2 Installation efficiency	0.0081
				2.4.3 Installation report	0.0081
				2.4.4 Installation service	0.0083
		2.5 Equipment commissioning and quality control	0.0327	2.5.1 Installation and commissioning	0.0086
				2.5.2 Quality inspection	0.0079
				2.5.3 Quality control record	0.0083
				2.5.4 Quality control service satisfaction	0.0080
		2.6 Equipment acceptance	0.0337	2.6.1 Acceptance process	0.0089
				2.6.2 Acceptance Time	0.0080
				2.6.3 Unacceptable processing	0.0084
				2.6.4 Acceptance service	0.0084
		2.7 Data protocol	0.0293	2.7.1 Data opening	0.0293
		2.8 Primary operational training	0.0327	2.8.1 Normative training	0.0107
				2.8.2 Clinical training	0.0111
				2.8.3 Medical training	0.0109
post-sale service	0.6087	3.1 Maintenance system	0.0445	3.1.1 Maintenance scale	0.0064
				3.1.2 Maintenance and certification	0.0068
				3.1.3 Maintenance system	0.0068
				3.1.4 Maintenance implementation normative	0.0069
				3.1.5 Maintenance response time	0.0076
				3.1.6 Troubleshooting time	0.0083
		3.2 Post-sales service personnel	0.0445	3.2.1 Post-sales team	0.0099
				3.2.2 Team training	0.0113
				3.2.3 Satisfaction with team service	0.0115
				3.2.4 Satisfaction with maintenance service	0.0118
		3.3 Complaint handling	0.0394	3.3.1 Complaint record	0.0099
				3.3.2 Complaint handling	0.0099
				3.3.3 Convenience of complaints	0.0102
				3.3.4 Complaint feedback	0.0102
		3.4 Adverse event monitoring	0.0408	3.4.1 Adverse event monitoring	0.0102
				3.4.2 Report of adverse events	0.0098
				3.4.3 Adverse event handling	0.0100
				3.4.4 Adverse event record	0.0108
		3.5 Maintenance and use manual	0.0459	3.5.1 Operation manual	0.0157
				3.5.2 Service manual	0.0152
				3.5.3 Openness of technical data	0.0149
		3.6 Maintenance response	0.0468	3.6.1 PM program	0.0096
				3.6.2 PM service satisfaction	0.0089
				3.6.3 Satisfaction with maintenance hotline	0.0087
				3.6.4 Satisfaction with Maintenance Response	0.0094
				3.6.5 Satisfaction with troubleshooting	0.0098
		3.7 Maintenance accessories	0.0459	3.7.1 Quality of maintenance accessories	0.0119
				3.7.2 Speed of arrival of repair accessories	0.0116
				3.7.3 Satisfaction with maintenance price	0.0117
				3.7.4 Satisfaction with payment method	0.0106
		3.8 Standby machine	0.0426	3.8.1 Whether to provide a standby machine	0.0218
				3.8.2 Satisfaction with standby service	0.0208
		3.9 Warranty contract	0.0449	3.9.1 Contract Integrity	0.0092
				3.9.2 Satisfaction with contract economy	0.0084
				3.9.3 PM Satisfaction in Contract	0.0088
				3.9.4 Satisfaction with contract indicators	0.0090
				3.9.5 Satisfaction with Contract evaluation	0.0087
		3.10 Maintenance and repair report	0.0435	3.10.1 Satisfaction with reporting quality	0.0222
				3.10.2 Satisfaction with report completion rate	0.0213
		3.11 Retraining of clinical operations	0.0412	3.11.1 Satisfaction with operational retraining	0.0412
		3.12 Retraining in clinical application	0.0417	3.12.1 Satisfaction with application retraining	0.0417
		3.13 Technical support	0.0422	3.13.1 Technical support	0.0422
		3.14 Maintenance quality	0.0449	3.14.1 Probability of the same fault occurrence	0.0226
				3.14.2 Satisfaction with equipment Performance	0.0223

### The weights and important scores of service level indicators

Applying the AHP procedure and the percentage method, the weight of each service level indicator was calculated and presented in Table 9. The indicators at the First Level are ranked as post-sale service (0.6087), in-sale service (0.2568), and pre-sale service (0.1345), highlighting post-sale service is the most valued by users and is most important to manufacturers or dealers. Within the post-sale service category, most sub-indicators carry equal weights, for example, maintenance system and post-sales service personnel both carry 0.0445 weights.

### Case study

At present, there is no standard to follow when designing an evaluation system to assess service level of a medical device. To test and verify the applicability of the proposed EIS, and gain deeper insights into the performance of EIS, a case study was performed. In light of the market share, brand awareness, and of the use of medical endoscopes in Chinese market, Olympus, Shanghai Aohua, and Shanghai Chengyun were selected as case companies to test and verify the proposed EIS. In this case study, 10 questionnaires were distributed to each manufacturer, 30 questionnaires were returned and 30 responses were valid. In this survey, the number of respondents was 30, 80% of them had bachelor’s degree or above, 70% had technical titles above intermediate level and the relevant working years were longer than 5 years. According to the survey results, the Aggregate Index scores were calculated using the EIS, with Olympus scoring 4.17, Shanghai Aohua scoring 3.72 and Shanghai Chengyun scoring 3.28. The results were consistent with the evaluation of medical endoscopy service level in the market.

## Discussion

The quality of medical endoscope service is an essential factor in market competition and an important link related to medical safety and patient safety. This research build a medical endoscope service level evaluation index system based on pre-sale, in-sale and post-sale service through Delphi method. It provided a tool to end users to choose ideal service providers, and a channel for service providers to identify options for service improvement. A few medical endoscope brands were selected for test and verify the developed EIS, and the results show that the system is applicable and useful, as evidenced that the final index scores obtained from the EIS system match the actual situation.

### Construction of three-level index for medical endoscopy service evaluation

The establishment of the medical endoscope service level EIS covered the whole life cycle of the service, including pre-sale, in-sale, and post-sales as the first-level indicators, and other indicators at the second-level and third-level. The inclusion, clustering or deletion of indicators were determined using a rigorous procedure, combining subject expert judgement from the Delphi method, and objective quantitative criteria. In this process, some indicators were deleted, which included pre-sale indicators at the Second Level, such as product display, demand demonstration, new technology promotion, sales system, etc. The deleted post-sale indicators included product recall, scientific research cooperation and functional development, etc.

But all the indicators under the category of in-sale were retained at the Second Level, and only a portion of the indicators at the Third Level were adjusted.

Pre-sale indicators were pertaining to promotion, display and sales performance of medical endoscope manufacturers in the Chinese market, which had more intersections with users of medical institutions. Although the decision of choosing medical endoscopes in the procurement process would be affected by the manufacturers marketing strategies, we chose to ignore the pre-sale marketing behavior as this area is less related to the quality or performance of medical endoscope.

Most of the post-sales indicators were retained, except scientific research and functional development cooperation. Although these indicators were prospective, the focus of medical endoscope users’ service evaluation was on the safety and effectiveness of medical performance, while the value-added service functions such as scientific research cooperation were not relevant to most medical endoscope users. It is worth mentioning that the in-sale indicators had been fully retained, and only a small adjustment was made to the in-sale indicators at the Third Level. This aspect shows that the service behaviour in the sale was generally recognized, and the indicator design was relatively accurate. On the other hand, it shows that although the sale takes the shortest time in the whole service process, it is still very important.

### The core role of post-sales service evaluation in medical endoscopy evaluation

In this study, 3 first-level indicators, 24 s-level indicators and 68 third-level indicators were formed, and their weights were calculated respectively. The weights assigned to the three indicators at the First Level were 0.13, 0.25 and 0.62 respectively. This shows that the industry has strong emphasis on the after sale service provided for medical endoscope. Based on the responses received in the first round of questionnaire, we also found that manufacturers paid less attention to post-sale service than hospitals, but paid more attention to pre-sale service than hospitals. The reason is obvious, as manufacturers value the sales side, but the hospital cares about the experience of application and performances. Medical endoscope manufacturers paid more attention to the pre-sale of the products and the communication with the customers during the sales process, in order to maximize the profit of selling [28]. When the medical endoscope breaks down during use, the manufacturer’s profit level drops due to maintenance or repair fee, and a potential of loss of clients. Therefore, the manufacturer paid more attention to the pre- and mid-term sales process of the product. When endoscopes fail to work, hospitals and medical staffs, as the disadvantaged groups of medical endoscope users, wish to receive timely response and service from manufacturers or maintenance parties. Therefore, they would pay more attention to the post-sales service of medical endoscopes [5, 29].

### Selection and information bias

The observational study design in this research means that selection bias and information bias are present to some degree, which is the limitation of this research. Selection bias stems from the selection of the expert group in the Delphi study, which limits the comparability between groups being studied. To reduce the impacts of selection bias, the sampling method used in choosing experts was random, and the professionals who met our pre-defined criteria had equal probability to be included in the study. Future work will expand the Delphi study to multiple expert groups, to further refine the configuration of the EIS.

The use of questionnaire helps to collect a wider range of perspectives, views, and opinions on the service level of medical endoscope. However, information bias may arise from self-reporting bias (such as social desirability, or recall bias), or inaccurate estimation. The questions asked in this research do not concern private or sensitive topics, and anonymity and confidentiality were guaranteed at the time of data collection, hence social desirability bias is less likely to be present in this study. To overcome recall bias, we defined the selection criteria to choose experts in the Delphi study, requiring these members to closely engage in medical endoscope application or production, therefore, these respondents were supposed to have up to date knowledge to evaluate the service level. To ensure internal validity of the collected responses and to minimize the impacts of inaccurate estimation, Cronbach’s α was calculated to check data reliability, and quantitative criteria was introduced to reassess the indicators. The next phase of study will involve surveys with a wider group of experts who will rate the service indictors. In additional to the use of statistical methods in checking validity and reliability, we will compare the survey data and the data from Delphi study with Technical reports or Users’ Evaluation reports on medical endoscopes, to examine the validity of the self-reporting instrument.

## Conclusions

In the process of establishing the service index system, the following characteristics of Delphi method were fully embodied: (1) the adequacy of resource utilization, as experts came from different manufacturers and industries, and they could make full use of their experience and knowledge; (2) the reliability of the net conclusions, benefiting from the back-to-back approach, each expert made his own judgment independently, without being affected by other complicated factors; (3) the unity of the net conclusion.

The EIS of medical endoscope established in this study, covers the whole life cycle of pre-sale, in-sale and post-sale of an endoscope; the EIS provides a comprehensive evaluation on the product (endoscope), from the aspects of manufacturers or service providers, as well as end user. A combination of qualitative and quantitative methods was applied to develop the EIS, combing subjective judgement and quantitative assessment. Therefore, the evaluation system constructed by the method of expert consultation has certain credibility and be applied to related fields. However, the results of this study are only carried out with a small group of samples, thus lack of testing in other problem settings. The research group plans to promote the application of the medical endoscope service level evaluation system in domestic medical institutions and manufacturers. Through iterative method and repeated expert methods, the evaluation system will be reviewed and upgraded to serve the national medical endoscope industry in the future.

## Supplementary information

**Additional file 1.** First round of questionnaire

## Data Availability

The datasets used during the current study are available from the corresponding author on reasonable request.

## Abbreviations

EIS: Evaluation Index System
AHP: Analytic hierarchy process
Cr: Degree of expert authority
Cs: Expert’s knowledge base to judge the program
Ca: Expert’s familiarity with the problem
M: Average Score
K: Full Score Frequency
Q+: The maximum value of the expert’s importance score
Q-: The minimum value of the expert’s score
Q + -Q-: The extreme value of the expert index score
