# Supplementary material for: Model construction of medical endoscope service evaluation system-based on the analysis of Delphi method
Source: BMC Health Serv Res. 2020 Jul 9;20:629. doi: 10.1186/s12913-020-05486-x (PMC7346529; doi:10.1186/s12913-020-05486-x)
Supplement: Supplementary file 1 — Additional file 1. First round of questionnaire [file 12913_2020_5486_MOESM1_ESM.doc]

**Appendix-1 First round of questionnaire**

**General information:** (1) employer information; (2) number of working years; (3) professional title; (4) positional title;

| **Note 1:** Importance score of service level indicator is rated at five levels, namely: very important / very good = 5; important / good = 4; moderately important / acceptable = 3; slightly important/ poor = 2; not important / very poor = 1. | | | | | | | | | |
| --- | --- | --- | --- | --- | --- | --- | --- | --- | --- |
| **Note 2:** please rate the familiarity and judgment basis of indicators. For example, if you are familiar with the indicator and the evaluation of its importance comes from authoritative data, please rate 5 in "familiarity score" and "judgment score"; if you are not familiar with the indicator or have no judgment basis, please rate 1 accordingly. | | | | | | | | | |
| **First level indicators** | **No.** | **Second level indicators** | **Importance score (1-5)** | **Familiarity score (1-5)** | **Judgement score (1-5)** | **Third level indicators** | **Importance score (1-5)** | **Familiarity score (1-5)** | **Judgement score (1-5)** |
| **1、Pre-sale service** | **1.1** | **Market system** |  |  |  | **1.1.1 Sales volume** |  |  |  |
|  |  |  |  |  |  | **1.1.2 Occupancy** |  |  |  |
|  | **1.2** | **Product display** |  |  |  | **1.2.1 In hospital training demonstration** |  |  |  |
|  |  |  |  |  |  | **1.2.2 Out of hospital training demonstration** |  |  |  |
|  | **1.3** | **Technical information** |  |  |  | **1.3.1 Technical information** |  |  |  |
|  | **1.4** | **Technical solutions** |  |  |  | **1.4.1 Providing technical solutions** |  |  |  |
|  |  |  |  |  |  | **1.4.2 programme completeness** |  |  |  |
|  | **1.5** | **Requirement demonstration** |  |  |  | **1.5.1 requirement demonstration** |  |  |  |
|  | **1.6** | **Dissemination of new technology** |  |  |  | **1.6.1 Technology update** |  |  |  |
|  |  |  |  |  |  | **1.6.2 Promotion enthusiasm** |  |  |  |
|  |  |  |  |  |  | **1.6.3 Accessibility** |  |  |  |
| **2、Sale service** | **2.1** | **Sales system** |  |  |  | **….** |  |  |  |
|  | **2.2** | **Configuration project** |  |  |  | **….** |  |  |  |
|  | **2.3** | **Timeliness of arrival** |  |  |  | **….** |  |  |  |
|  | **2.4** | **Installation manual** |  |  |  | **….** |  |  |  |
|  | **2.5** | **Device installation** |  |  |  | **….** |  |  |  |
|  | **2.6** | **Equipment commissioning and quality control** |  |  |  | **….** |  |  |  |
|  | **2.7** | **Equipment acceptance** |  |  |  |  |  |  |  |
|  | **2.8** | **Data protocol** |  |  |  | **….** |  |  |  |
|  | **2.9** | **Primary operational training** |  |  |  |  |  |  |  |
|  | **2.10** | **Primary application training** |  |  |  |  |  |  |  |
|  | **2.11** | **Maintenance training** |  |  |  |  |  |  |  |
| **3、post-sale service** | **3.1** | **Maintenance system** |  |  |  | **….** |  |  |  |
|  | **3.2** | **Post-sales service personnel** |  |  |  | **….** |  |  |  |
|  | **3.3** | **Complaint handling** |  |  |  | **….** |  |  |  |
|  | **3.4** | **Adverse event monitoring** |  |  |  | **….** |  |  |  |
|  | **3.5** | **Product recall** |  |  |  | **….** |  |  |  |
|  | **3.6** | **Maintenance and use manual** |  |  |  | **….** |  |  |  |
|  | **3.7** | **Maintenance response** |  |  |  | **….** |  |  |  |
|  | **3.8** | **Maintenance accessories** |  |  |  | **….** |  |  |  |
|  | **3.9** | **Standby machine** |  |  |  | **….** |  |  |  |
|  | **3.10** | **Warranty contract** |  |  |  | **….** |  |  |  |
|  | **3.11** | **Adverse event handling** |  |  |  | **….** |  |  |  |
|  | **3.12** | **Maintenance and repair report** |  |  |  | **….** |  |  |  |
|  | **3.13** | **Retraining of clinical operations** |  |  |  | **….** |  |  |  |
|  | **3.14** | **Retraining in clinical application** |  |  |  | **….** |  |  |  |
|  | **3.15** | **Technical support** |  |  |  | **….** |  |  |  |
|  | **3.16** | **Customer complaint handling** |  |  |  |  |  |  |  |
|  | **3.17** | **Research cooperation** |  |  |  |  |  |  |  |
|  | **3.18** | **Function development** |  |  |  | **3.18.1 Function development** |  |  |  |
